# On the importance of field driven single particle processes in short pulse absorption of clusters

**DOI:** 10.1038/s41598-019-51385-5

**Published:** 2019-10-22

**Authors:** Soubhik Sarkar, R. Gopal, M. Anand, M. Praveen Kumar, M. Krishnamurthy

**Affiliations:** 1 Tata Institute of Fundamental Research, 1 Homi Bhabha Road, Colaba, Mumbai 400 005 India; 2 Tata Institute of Fundamental Research, Gopanpally, Hyderabad 500 107 India

**Keywords:** Macromolecules and clusters, Laser-produced plasmas

## Abstract

Nano-clusters are acclaimed to be very efficient absorbers of intense femto second light due to dominant collective mechanisms. Enhanced near 100% absorption due to collective linear plasma resonance compared to a small fraction of absorption by unclustered gas was an important drive in nano-plasma studies. Contrary to such perception, we show that if the pulse duration is (<100 fs), absorption is same irrespective of whether the systems are condensed to large clusters or not. So long as there are same number of similar ionizable systems in the focal volume, absorption is the same and such absorption can be accounted for by single particle response to the field and collisional ionization of atoms. Short pulse absorption by the single particle response can be comparable to the linear plasma resonance absorption for smaller clusters.

## Introduction

The interaction of high intensity(>10^15^ W/cm^2^) ultra short laser pulses with matter have been a novelty due to their ability to generate intense burst of ions, electrons and x-rays^1^. Of these, the study with matter in the form of nanoclusters^2–4^ has its own special place. Having the unique features of solid-like local density confined to dimensions smaller than the laser wavelength and absence of the usual energy dissipation channels of bulk condensed media, allows one to study intense laser plasma physics with much lesser complexity. A combination of these properties result in high absorption^5^, highest charge densities in the plasma^6,7^, MeV ions with only keV electrons^8,9^, sub-ps bursts of hard x-rays^10^ and even acceleration of neutral atoms and negative ions^11,12^.

A near 100% absorption when the Xe or Ar gas atoms is condensed to nano-clusters as compared to Ne or He that form very small or no-clusters has driven the nano-plasma science^5^. Current understanding of the interaction of intense laser pulse with clusters based on many important studies in this field can be summarised into the following steps: (a) Intense laser pulse ionizes atoms by field ionization (multi-photon, tunnel and over barrier ionization) (b) Electrons released from atoms are driven by the laser field and contribute to collisional ionization. (c) Increase in electron density leads to formation of a plasma and collective absorption mechanisms like linear plasma resonance begins to enhance absorption of light. (d) Cluster expands/explodes both under hydrodynamic^13^ or Coulomb^14,15^ pressure finally releasing high energy ions and electrons. The first two steps contribute to the absorption of the laser light by single particle response to the external field and is referred to as the ‘non-collective’ absorption mechanism in this paper. The only major collective absorption mechanism we refer to in this paper is the linear plasma resonance mentioned in step-(c). Here resonant matching of the laser and local plasma frequency lead to boosting the local electric field and an enhanced energy transfer from the electromagnetic field to the plasma fields by increased charge separation and electon-ion collisions. This is referred to as collective because the local field inside the cluster is a function of the dielectric constant, a bulk property.

**Figure 1:**
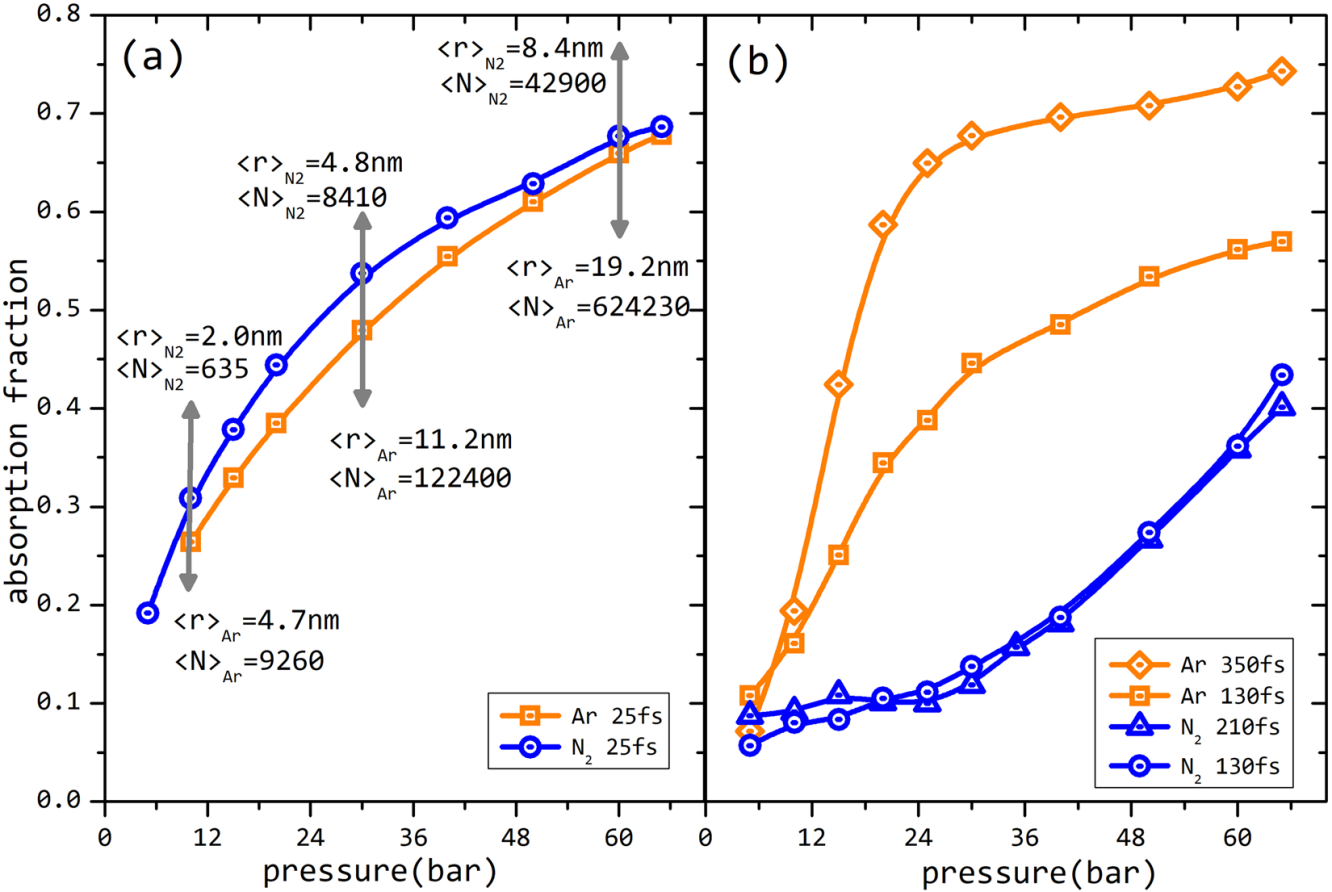
Absorption with cluster size: In (**a**) we see that with the shortest pulse of 23 fs, absorption for Ar and N_2_ is comparable irrespective of the fact that Ar forms bigger clusters and N_2_ forms considerably smaller clusters. The average cluster radius 〈r〉 and the average number of atoms per cluster 〈N〉 for the two gases is indicated for a few representative pressures by the arrows. In (**b**) we see that with the longer pulses the absorption with N_2_ and Ar are very different. Ar shows a much steeper raise in absorption with pressure. Longer pulses demonstrate signatures of linear plasma resonance while absorption in short pulses is dominated by single particle response (see text).

While this simple picture is appealing^2–4^, still many questions remain for want of answers. Are collective mechanisms important at all for nano-clusters with very short laser pulse interaction? Is absorption by an enhanced local field, a result of collective mechanism of the nanoplasma, the only way of large energy deposition in laser cluster interactions? Answer to many to these questions are often sought by computational models^3,4^. Particle-in-cell simulations or analytical computation schemes are used to switch on or switch off the collective mechanisms and the role of the laser energy absorption in the different steps is sought. In the many experiments performed in these systems, it appears that there is no clear strategy to experimentally differentiate absorption due to non-collective processes from collective absorption. We demonstrate in this paper, that a comparative study of laser absorption using Ar and N_2_ as target gases under the same conditions, provides a clear strategy to probe some of these important questions. We clearly establish that for 800 nm pulses shorter than 100 fs and having pulse energies of ~3.0 mJ focussed to intensities ~10^16^ W/cm^2^, absorption is predominantly by single particle response to the external field (steps a and b mentioned above). Absorption for short pulses is the same so long as the same number of atoms/molecules are present in the focal volume irrespective whether they are bound into van der Waals clusters or not. The short pulse absorption is dominated by the single particle response which itself can be as large as that due to linear plasma resonance. Only for longer pulse duration and larger clusters, does the effects of collective mechanisms have any significant contribution in influencing the absorption of energy (see Fig. 1b).

## Results and Discussion

Conventional wisdom in the nano-clusters studies covers pulse durations larger than 100 fs. On this timescales one would expect a larger absorption in a cluster aggregates^5^ compared to poorly aggregated gas due to the occurrence of collective plasma resonance. An attempt to address the question whether this is applicable for short pulses or not, is presented in Fig. 1. In Fig. 1(a) which shows the absorption of 23 fs pulse with Ar and N_2_ as a function of the pulsed valve backing pressure, it is clear that there is little difference in absorption between the two. Small differences in absorption between Ar and N_2_ also become even smaller as the total number of atoms in the focal volume is increased. Absorption in this case is only dependent on the pressure (monotonic increase) and ability of Ar to form bigger clusters makes little difference to the absorption. It appears that the absorption is only dependent on the number of atoms of Ar and N_2_ present in the focal volume. Since, the ionization potentials for the two gases are very similar (one reason why they were chosen in the first place) and the number of the atoms in the focal volume is only dependent on pressure, same absorption for such two gases can then be comprehended if the absorption is predominantly due to the single particle response to the external field with little influence of collective behaviour.

Ar and N_2_ has similar first few ionization energies. The first two ionization potentials for Ar are 15.75 eV and 27.69 eV and for N_2_ they are 15.58 eV and 27.9 eV, respectively^16^. Higher ionization potentials computed for N_2_ by the dissociation limits of the atomic ions are slightly higher than that of Ar. Ionization to these states are by barrier suppression or by field driven electron impact ionization. The thresholds for barrier suppression to a 7+ charge state for Ar is about 2.0 × 10^16^ Wcm^−2^ and it is 2.6 × 10^16^ Wcm^−2^ for N_2_. So it would make little difference in ionizability between N_2_ and Ar, especially for the intensities used here. Computation of the cross sections for electron impact ionization accounted by Lotz formalism^17^ show that the cross section for ionization of Ar and N_2_ for 1.2 keV electrons (ponderomotive energy at 2.0 × 10^16^ Wcm^−2^) differ between 15–20%. So if the absorption is due to field driven single particle response (multiphoton, tunnel, over-barrier and field driven electron impact ionization), Ar and N_2_ should be little different, irrespective of the fact that Ar is in the form of clusters while N_2_ is expected to only have a small fraction of much smaller clusters. Similar absorption with N_2_ and Ar shown in Fig. 1(a) irrespective of the backing pressure is an experimental proof of this inference. To establish that this absorption is equally as strong but different from the collective phenomena dominant at longer pulse duration, measurements shown in Fig. 1(b) is in order. In Fig. 1(b) the same exercise is repeated but for longer pulse durations. Although the absorption values for Ar as a function of pressure are roughly only as large as that in Fig. 1(a), there is a stark difference between the values and the nature of the curve when compared between Ar and N_2_, unlike that in Fig. 1(a). This indicates that the dominant processes in this regime is not the same as that for the short pulses(~25 fs). Absorption for Ar is seen to be much more, increasing much more steeply as compared to N_2_ reaching a saturation value. It can also be seen that as the pulse width is made larger, the absorption increases even more steeply and saturation is attained with even smaller size clusters or at a lower backing pressure. This and the absence of such a trend in N_2_ is indicative of collective mechanisms at play in this regime because while Ar forms larger clusters, the poorly aggregated N_2_ molecules do not allow for such processes and fail to reach the collective resonance leading to increased absorption.

**Figure 2:**
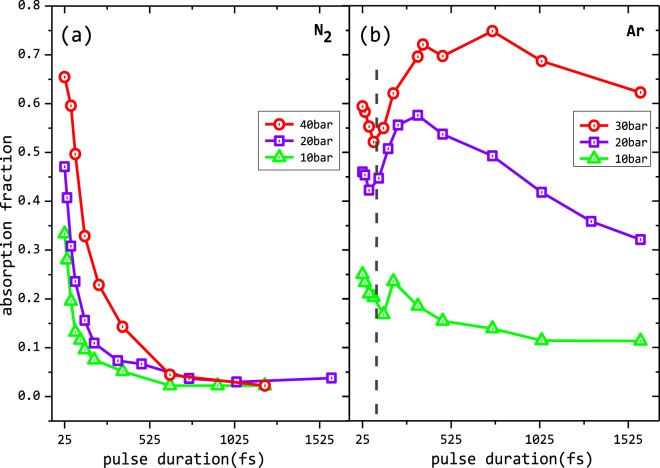
Absorption with pulse duration: Absorption of the laser energy with the change in pulse duration at a constant laser energy for (**a**) N_2_ and (**b**) Ar clusters at different backing pressures, respectively. At shortest pulse, the absorption is similar. As the pulse duration increases, N_2_ shows a monotonic decrease in absorption, whereas Ar exhibits signature due to linear plasma resonance. Mean cluster sizes are indicated. Dotted line points to the minima in absorption and the reversal of increase in absorption for shorter pulses.

In order to present the complete picture of the transition from one regime to another, a scan of the pulse durations was conducted to measure absorption in Ar and N_2_ as a continuous function of pulse width. This is presented in Fig. 2 which exhibits both the similarities and differences between the two. Irrespective of the backing pressure, for the N_2_ clusters there is a systematic reduction in absorption with increase in pulse width. However, for Ar a different feature appears. For very short pulses (23–100 fs) the absorption is similar to that of N_2_. Absorption decreases till about 100 fs but after that there is a reversal in the trend and absorption increases again with increase in pulse width. Depending on the cluster size, the absorption increases to a peak value and further increase in pulse width decreases the absorption. The raise in absorption, peaking to large value depending on the cluster size and decrease further, is a well known feature of linear plasma resonance in clusters. This has been reported before with laser pulses of longer duration^5^ and is comprehended in many computation models^4,18^. It is attributed to the occurrence of a resonant coupling of the laser light with the collective oscillatory motion of the electrons having a characteristic natural frequency, the local plasma frequency. The field inside the cluster nanoplasmas that are much smaller in size as compared to the laser wavelength can be described as^2,5^

1 $$E=\frac{3}{|\varepsilon +i\nu |}{E}_{0}$$

where *ε* and *v* are the plasma dielectric constant and the electron-ion collision frequency respectively and E_0_ is the laser electric field. With the internal electric field defined as above, the rate of energy deposition in the clusters, can according to the Poynting’s theorem, be written in terms of the rate of change of the energy density U as,

2 $$\begin{array}{rcl}\frac{\partial U}{\partial t} & = & \frac{1}{4\pi }{\bf{E}}\cdot \frac{\partial {\bf{D}}}{\partial t}\\  & = & \frac{9{\omega }^{2}{\omega }_{p}^{2}\nu }{8\pi }\frac{1}{9{\omega }^{2}({\omega }^{2}+{\nu }^{2})+{\omega }_{p}^{2}({\omega }_{p}^{2}-6{\omega }^{2})}|{E}_{0}{|}^{2}\end{array}$$

where the relation between the plasma dielectric constant *ε* and the plasma frequency *ω*_*p*_,

3 $$\varepsilon =1-\frac{{\omega }_{p}^{2}}{\omega (\omega +i\nu )}$$

has been used. Model calculations using this formalism have been studied and Fig. 8 given in review^3^ gives a good exposition of this plasma resonance scheme. Owing to the much slower motion of the ions and the trapping of the quasi-free electrons in the potential of these background ions, the initial ionization of the cluster medium generates an almost solid density plasma with an n_*e*_ close to 10^23^ cm^−3^ and thus a plasma frequency in the ultra-violet. But as the hydrodynamic forces expand the clusters over a few hundred fs(s) and the n_*e*_ decreases to 3n_*c*_, where n_*c*_ is the critical density for 800 nm, both the field inside the cluster nano-plasma as well as the rate of energy deposition reaches a maximum. This can be easily verified by plotting Eq. 2 as a function of n_*e*_/n_*c*_. This resonance clearly is a collective phenomena as indicated by the inclusion of collective properties like the dielectric constant in the equations above. The energy deposited due to these processes should also likewise be dependent on the size of the collective systems. Thus linear plasma resonance heating mainly due to the inverse Bremsstrahlung processes^4^ gives the maximum absorption.It should be kept in mind that the maxima of absorption due the linear plasma resonance and the resonance widths are strong functions of the laser intensity, wavelength and the cluster size. The physics issues brought out in this papers are limited to smaller clusters (<1/10 of the laser wavelength) and in the non-relativistic intensity regime (10^15–17^ Wcm^−2^). However if the cluster expansion is too slow, it is possible that the critical density is not reached before the end of the laser pulse and there is no resonance absorption. For smaller clusters, formed especially with N_2_, this resonance mechanism does not appear to affect the absorption significantly as it does for the larger Ar clusters at longer pulse duration. The expansion velocity in coulomb explosion depends on both the charge (average charge/atoms times the number of atoms/cluster) and mass of the atomic systems. Smaller cluster with smaller total Coulomb charge will expand slowly, even if the mean charge per atom is the same. For the same backing pressure, the energy of N is several times smaller than that of Ar.^7,19^. As a result, N_2_ clusters do not exhibit collective behaviour for the range of pulse duration studied here. This is clearly seen in the data presented in Fig. 2(a) where we do not see increase in absorption with increase in pulse duration for N_2_ while for Ar in Fig. 2(b), as the cluster size increases, the linear plasma resonance feature increases and begin to dominate the absorption at longer pulse duration.

A reversal and increase in absorption as the pulse duration is made shorter than 100 fs, not withstanding the changes in the intensity, is contrary to the expectations of linear plasma resonance absorption in clusters and has not been reported in contemporary literature. Short pulse absorption clearly depend only on the number of atoms present in the focal volume and can be attributed to the field dependent ionization of atoms, without the influence and modifications due to collective properties. Possibilities of anharmonic resonance due to increase in intensities^20^ could also be safely disregarded because of the similitude of the absorption values between the smaller N_2_ and larger Ar clusters as observed in this regime.

**Figure 3:**
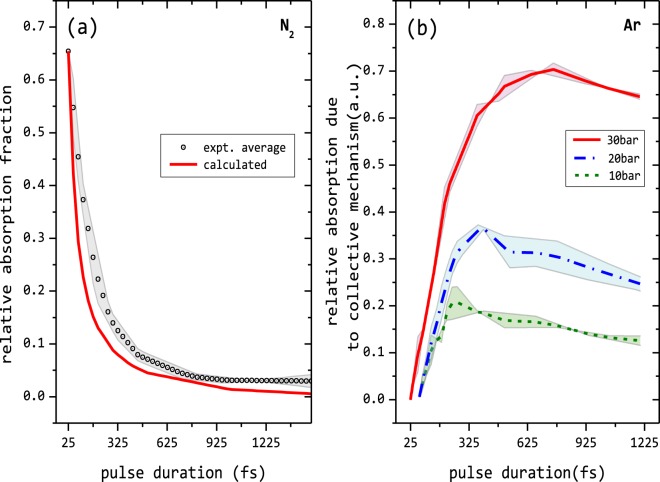
Absorption by collective and non-collective mechanisms (**a**) Absorption in N_2_ clusters with pulse duration is modelled taking into account ionisation by tunnelling, over the barrier ionisation and field driven collisional ionization computed by Lotz formalism. The calculated absorption curve closely follows the experiments. (**b**) Relative absorption fraction deciphered solely to be due to collective mechanisms for different cluster sizes. The shaded region about the lines gives the error.

A computation of the absorption by field ionization is presented in Fig. 3(a). We use the gaussian pulse propagation formalism to first compute the spatial dependent intensity. The effects of the variation of the laser intensity over the focal volume is taken such that the low intensity regions both spatially and temporally may be transparent or contribute to only small fraction of the absorption. As the pulse intensity increases beyond 10^10^ Wcm^−2^, ionization is possible by tunnel ionization. The probability of ionization of an atom at a given position is computed using ADK (Ammosov, Delone, and Krainov) tunnel ionization which is given to be to be^21^

4 $$W({E}_{s})=4\omega {({I}_{p})}^{5/2}E\,\exp [-{(2{I}_{p})}^{3/2}3E]$$

where *ω* is the atomic frequency, E is the laser electric field, and I_*p*_ is the ionization energy. At higher intensities, beyond the over barrier ionization threshold^22^ we use the direct ionization rate. The corresponding energy from the field is depleted so that in the next spatial slice of the laser pulse propagation is at lower laser intensity. The electrons generated at that point is assumed to be ponderomotively accelerated by the field and energy dispersed in collision ionization is calculated using the lotz formula^17^. The energy transferred to the atom by laser drive electron-impact ionization is again depleted. In the next spatial slice the laser pulse propagates with the corrected reduced intensity and similar computation is repeated. At the end of the propagation, the energy remaining in the field gives the unabsorbed light (I^*t*^) transmitting out of the focal area. Absorbance is computed as (I^0^ − I^*t*^)/(I^0^). Thus the total absorption of light is computed by integrating the fractional absorption through the different intensity regions and also accounting for loss in propagation through each cross-section.

As the pulse duration increases, the intensity decreases and the absorption due to the field ionization decreases. Change in the absorption due to collisional ionization due to the fact that N_2_ has small clusters is also incorporated by taking the appropriate electron density the clusters expand in time. To account for cluster expansion the hydrodynamic expansion rate has been considered with the average electron temperature taken to be about the ponderomotive energy.The experimental measurements for N_2_ at a pulse valve backing pressure of 40 bar is normalised with the computed absorption for the shortest pulse as shown in Fig. 3(a). The computations compare well with the measurements and the similarity in the trend in Fig. 3(a), it is re-affirmed that short pulses interacting with small cluster like N_2_ can be effectively modelled as systems where the primary mode of light absorption is only due to the single particle - field responses: multi-photon ionization, over the barrier ionization and field driven electron impact ionization. We note that the computed absorption for pulse duration around 100 fs is smaller than that from experiments. The model that assumes only atomic ionisation processes does not include collective contributions like for example, there could be longitudinal drift of electrons as the laser propagates. For the experimental conditions used, there will be small N_2_ clusters and at larger pulse duration there will be some contribution to the absorption by collective mechanisms. The model that does not account for these effects is expectedly shows smaller absorptions than the experimental measurements. This difference is evidently much smaller in the very short and very long pulse durations where collective effects should not make much difference. Till about 100 fs where the absorption becomes minimal (See dotted line of Fig. 2(b)) the absorption even in Ar, which follows closely to that of N_2_, is dominated only by single particle response and there is very little absorption due to the collective mechanisms. We can use thus use this relative difference in the absorption of Ar and N_2_ from Fig. 2 to obtain an experimental measure of the absorption solely due to the collective linear plasma resonance mechanism. The plots given in Fig. 3(b) gives these results.

Also as is seen, for smaller clusters absorption due to collective mechanisms is comparable to the absorption due to single particle response. Figure 2(b) shows that for Ar 10 bar (<N>_*Ar*_ ≃ 9250) absorption at 25 fs is same as it is at 250 fs. Larger the cluster, stronger is the absorption due to collective mechanisms. At 30 bar or <N>_*Ar*_ ≃ 122, 500 atoms, absorption at 25 fs is 60% as compared to 75% at 700 fs. The shaded region in the Fig. 3(a,b) denote the error in measuring the experimental curves of absorption for N_2_ and Ar respectively.

So, although the linear plasma response phenomenon is understood there are difficulties in comprehending the magnitude of absorption. An expanding nano-plasma passes the resonance point for a very short duration and power transferred to the cluster ensemble is not large enough to account for the x-ray/electron emission^13^. So models with non-uniform density are proposed such that the resonance condition is met for longer duration as different radial expansion zones come into resonance. It may be anticipated such a non-uniform expansion feature should also work for shorter pulse duration and there should have been signatures of resonance absorption even with shorter pulses. The experiments shown here does not support this possibility. For pulses shorter than the 100 fs and in the parameter space explored here, absorption is characteristically different and seem understandable well by single particle response to the external field. We note that the short pulses here in fact have higher intensity so the ability to produce stronger ionization is only larger. For clusters upto about 5 nm, absorption due to single particle response is comparable to that from the collective mechanisms in a direct experimental setting. We also note that recently theoretical simulations in the 5 fs pulse regime^20^ have demonstrated the possibility of frequency shifts in anharmonic plasma resonance absorption. The present experiments used longer pulse durations $\geqq$23 fs, which limits the applicability of these previous results and the potential to measure them. Instead, single particle processes play the central role in laser absorption in this regime, as described in this paper.

To summarize, intense ultrashort pulses impinging on matter are absorbed to start with by single particle response to field like multi-photon ionization, tunnel ionization or over the barrier ionization. Collisional ionization by the field driven electron further contribute to absorption by single particle response. Once the electron density increases and if the plasma frequency matches with the laser frequency, linear plasma resonance enhances the laser absorption. Our experiments show that in nano-clusters upto a size of about 14 nm, for pulse width shorter that 100 fs, absorption is predominantly due to the single particle response and there is a very little contribution to absorption by collective mechanism. Larger pulse duration with Ar clusters do show linear plasma resonance effect very similar and in concurrence to the previous experiments^5^. N_2_ with very similar ionizability as Ar works well as a system to compare the absorption due to collective and non-collective mechanisms.

## Methods

The experiment is performed with an 800 nm 3.5 mJ laser having a 1 kHz pulse repetition rate and generates shortest pulses of 23 fs that are focused onto a cluster jet using an off-axis parabolic mirror to achieve 5 × 10^16^ Wcm^−2^ intensity. The cluster jet is produced using a pulsed solenoid valve fitted with 500 *μ*m conical nozzle. The mean size of the clusters in the beam is systematically varied by varying the backing pressure of the pulsed solenoid valve. The experiments are carried out in the regime where there is near 100% clusterisation with Ar and clusterisation is about 86% in case of N_2_^23^. The cluster systems are characterised by Rayleigh scattering experiments carried out on this supersonic jet system earlier^24^.The mean cluster radius 〈r〉 and number of atoms per cluster 〈N〉 for the gases used Ar and N_2_ was estimated using the Hagena scaling parameter Γ^*^^25^. The pulsed valve is operated with a repetition rate of about 3 Hz and is synced to operate with the laser so that every gas pulse would interact with one laser pulse. We compare the signal level of the laser pulse that interacts with the cluster jet and those that does not, using a photo-diode placed after the region of interaction. The scattering losses are less than 5% as measured by a photodiode calibrated to a power meter. The average reduction in transmission as measured over $\gtrsim 200$ shots per data point gives the fraction of the laser energy absorbed by the cluster medium. Pulse width is varied by changing the separation distance between the pair of transmission type compressor gratings used to temporally compress the laser pulse. Pulse duration is measured using an auto-correlator for short pulse durations. For longer pulse durations we use the dispersion parameters to compute the pulse duration.

The key strategy in this experiment is the choice of the gases made for comparison. Both Ar and N_2_ have very similar ionization potentials. Ar with larger polarizability forms large clusters even at low pressure in the backing chamber while on the other hand N_2_ has very low polarizability and does not efficiently form clusters. At any given backing pressure and identical operating conditions (nozzle size, pulse valve opening time etc.), the number of the atoms in the focal volume would be same except that in the case of Ar there would be nano-clusters and in the case of N_2_ it would be nascent molecules along with a smaller number of smaller clusters. Absorption of light in these two systems is the measure that we use to probe the role of collective mechanisms or the lack of it.
